# Diverse modes of genomic alteration in hepatocellular carcinoma

**DOI:** 10.1186/s13059-014-0436-9

**Published:** 2014-08-26

**Authors:** Suchit Jhunjhunwala, Zhaoshi Jiang, Eric W Stawiski, Florian Gnad, Jinfeng Liu, Oleg Mayba, Pan Du, Jingyu Diao, Stephanie Johnson, Kwong-Fai Wong, Zhibo Gao, Yingrui Li, Thomas D Wu, Sharookh B Kapadia, Zora Modrusan, Dorothy M French, John M Luk, Somasekar Seshagiri, Zemin Zhang

**Affiliations:** Department of Bioinformatics and Computational Biology, Genentech Inc., South San Francisco, CA 94080 USA; Department of Molecular Biology, Genentech Inc., South San Francisco, CA 94080 USA; Department of Infectious diseases, Genentech Inc., South San Francisco, CA 94080 USA; Department of Pathology, Genentech Inc., South San Francisco, CA 94080 USA; Department of Surgery, University of Hong Kong, Pokfulam, Hong Kong; BGI-Shenzhen, Shenzhen, 518083 China; Department of Pharmacology, National University of Singapore, Singapore, 117597 Singapore; Institute of Molecular and Cell Biology, A*STAR, 61 Biopolis Drive, Singapore, 138673 Singapore

## Abstract

**Background:**

Hepatocellular carcinoma (HCC) is a heterogeneous disease with high mortality rate. Recent genomic studies have identified *TP53*, *AXIN1*, and *CTNNB1* as the most frequently mutated genes. Lower frequency mutations have been reported in *ARID1A*, *ARID2* and *JAK1*. In addition, hepatitis B virus (HBV) integrations into the human genome have been associated with HCC.

**Results:**

Here, we deep-sequence 42 HCC patients with a combination of whole genome, exome and transcriptome sequencing to identify the mutational landscape of HCC using a reasonably large discovery cohort. We find frequent mutations in *TP53*, *CTNNB1* and *AXIN1*, and rare but likely functional mutations in *BAP1* and *IDH1*. Besides frequent hepatitis B virus integrations at *TERT*, we identify translocations at the boundaries of *TERT*. A novel deletion is identified in *CTNNB1* in a region that is heavily mutated in multiple cancers. We also find multiple high-allelic frequency mutations in the extracellular matrix protein LAMA2. Lower expression levels of *LAMA2* correlate with a proliferative signature, and predict poor survival and higher chance of cancer recurrence in HCC patients, suggesting an important role of the extracellular matrix and cell adhesion in tumor progression of a subgroup of HCC patients.

**Conclusions:**

The heterogeneous disease of HCC features diverse modes of genomic alteration. In addition to common point mutations, structural variations and methylation changes, there are several virus-associated changes, including gene disruption or activation, formation of chimeric viral-human transcripts, and DNA copy number changes. Such a multitude of genomic events likely contributes to the heterogeneous nature of HCC.

**Electronic supplementary material:**

The online version of this article (doi:10.1186/s13059-014-0436-9) contains supplementary material, which is available to authorized users.

## Background

Hepatocellular carcinoma (HCC) is the third leading cause of cancer-related death, with a poor 5-year survival rate of less than 10% [1]. While more than 600,000 new cases are diagnosed annually, there is no effective targeted therapy. HCC is highly heterogeneous and associated with various etiological factors, including hepatitis B virus (HBV) or hepatitis C virus (HCV) infection, alcohol consumption and exposure to aflatoxin and possibly vinyl chloride [2]. Several emerging themes were revealed by recent genomic studies [3–6], including recurrent mutations in *TP53*, Wnt-signaling components *CTNNB1* and *AXIN1*, and chromatin regulators like *ARID1A* and *ARID2*, as well as HBV integration near *TERT*, *CCNE1* and *MLL4*. The frequently altered genes discovered by these studies have differed, however, possibly due to small discovery panels (up to 25 patients) and the inherent heterogeneity of HCC due to several associated etiological factors. For example, activating mutations in *CTNNB1* are mutually exclusive with HBV infection [6] and hence would not be prominent in a cohort enriched for HBV-infected patients. Therefore, it might not be surprising that different studies have identified different genes that are mutated in the population at low frequency, like *ARID2*, *ARID1A* and *JAK1*. Another difficulty in constructing the complete mutational landscape in HCC is the focused nature of most previous studies, since it is not common for a single study to comprehensively examine multiple types of genomic changes that include point mutations, deletions, structural variations, and virus-mediated mutations. We have previously shown that HBV DNA frequently integrates into the human genome, causing diverse changes such as DNA copy number variation, chimeric viral-human transcript fusions, and transcriptional activation [3]. Given the disruptive nature of HBV integration, it is pertinent to study all modes of genomic changes in the same context.

In this study, we analyzed a panel of 42 HCC patients with a combination of whole-genome, exome and transcriptome sequencing. We identified multiple high-allelic frequency mutations in *LAMA2. LAMA2* encodes the α subunit of laminin, the major component of basal laminae. Besides being a structural component of the extracellular matrix, basal laminae can influence cell proliferation and differentiation. Defective anchoring to laminins is widespread in cancer [7]. The high incidence of mutations in an extracellular matrix component like LAMA2 adds a new dimension of underlying genetic components to this rather complex disease. We also found two patients with mutations in *IDH1* at the R132 hotspot found in other cancers [8,9], and one patient with truncated *IDH2*. The tumor suppressor *BAP1* was mutated in two patients. We previously showed that HBV randomly integrates into the human genome and results in several genomic and genetic alterations [3]. Here we report HBV integrations in eight HBV-infected patients, including integration in the vicinity of three previously reported genes, *TERT*, *CCNE1* and *MLL4*. Aside from individual genes mutated in HCC, we also examine the diverse modes of genomic alteration in this heterogeneous disease, delineating both conventional mutations and virus-associated changes that contribute to liver oncogenesis.

## Results

### The mutational landscape of hepatocellular carcinoma

We analyzed 42 HCC tumor-normal pairs to identify frequent and high-allelic frequency mutations. We sequenced whole genomes and transcriptomes of 12 patients, and exomes of 30 additional patients. We identified 49 non-silent mutations per patient (median value; Table S1 in Additional file 1; Figure S1A in Additional file 2) from the 12 whole genomes, and 54 non-silent somatic mutations per patient (median value; Table S2 in Additional file 1; Figure S2 in Additional file 2) from the 30 exomes. One of the patients had an unusually high mutation rate (Figure S2 in Additional file 2), with >99% of the point mutations of the C > T type (Table S2 in Additional file 1). The mechanism of such a high mutation rate in this particular patient is not clear, but such a C > T mutation pattern is consistent with APOBEC3B-mediated mutagenesis observed in other types of cancers [10,11].

**Figure 1:**
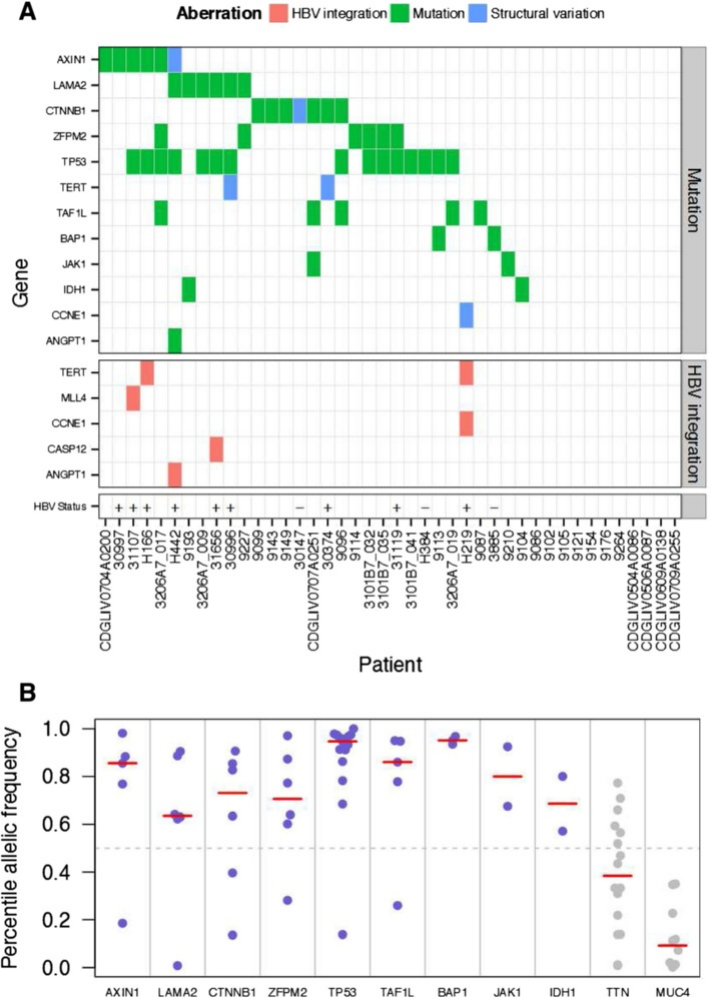
**Summary of genomic aberrations in hepatocellular carcinoma. (A)** Select genes that have multiple point mutations at high allelic frequency or structural variations are shown. Select cancer gene census genes that showed mutations at high allelic frequency and highly clonal viral integrations are also shown. HBV infection status was known for the 12 whole-genome sequencing samples. For these samples, HBV infection status is shown as a plus sign if infected, and a minus sign if no infection was detected. **(B)** Allelic frequency profiles of frequently mutated genes across whole genomes and exomes. For each mutant allele found in the study, the percentile of its allelic frequency (calculated for each sample separately) is shown on the y-axis. The red bars indicate the median value of the percentile allelic frequency. Blue dots represent chosen mutations, where the median percentile value was more than 0.5, while the grey dots show examples of genes that had median values below 0.5 and were rejected.

**Figure 2:**
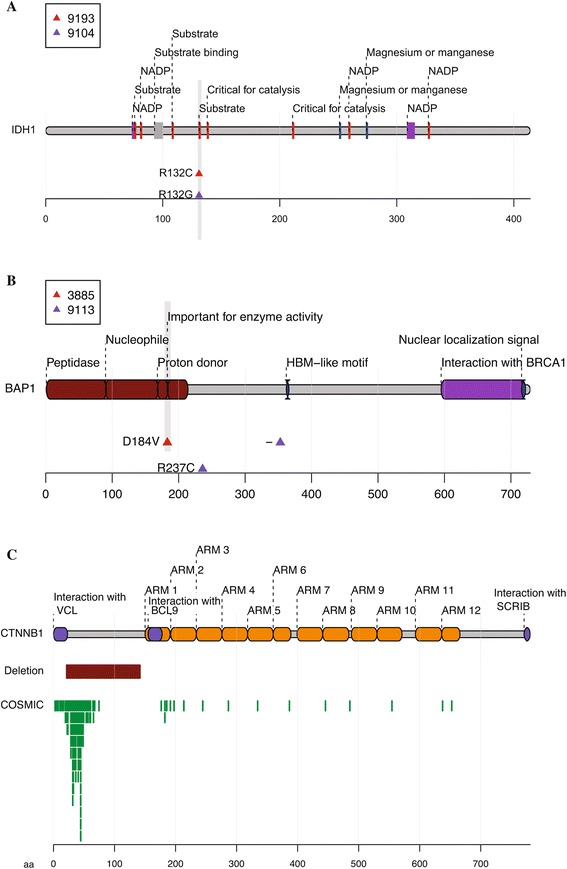
**Mutations in** ***IDH1*** **,** ***BAP1*** **and** ***CTNNB1*** **. (A)** *IDH1* is mutated in two patients at the hotspot R132. **(B)** *BAP1* mutation in two patients. Patient 3885 has a D184V mutation, a site that is important for the peptidase enzymatic activity (by homology). The other patient (9113) carries two mutations, including a point mutation, R237C, and a frameshift deletion at position 354. **(C)** An in-frame deletion in *CTNNB1* in patient 30147. The deletion spans 121 amino acids near the amino-terminal. This region is involved in the degradation of β-catenin and is frequently mutated in cancer, as shown by clustering of mutations in this region in the COSMIC database. Each green box is a mutation instance in COSMIC. Overlapping, unique mutations have been stacked.

We found frequent non-silent mutations in *TP53*, *CTNNB1*, *AXIN1*, *LAMA2*, *ZFPM2* and *TAF1L* (Figure 1A). We focused on mutations identified at high allelic frequencies (Figure 1B), as they are likely to be functional. Frequent mutations in *TP53*, *CTNNB1* and *AXIN1* have been reported previously in HCC, and these occur in 13 out of 42 patients (Figure 1A). Additionally, we found mutations in *IDH1* at codon 132 (2/42 cases; Figure 2A), a hotspot for mutations in glioblastoma and intrahepatic cholangiocarcinoma [9], thus expanding the indications for *IDH1* mutations. Mutations at codon 132 in *IDH1* have been shown to dominantly inhibit the catalytic activity of *IDH1*, which normally functions to catalyze the oxidative decarboxylation of isocitrate to α-ketoglutarate, and contribute to tumorigenesis by inducing the HIF-1 pathway [12]. Interestingly, another patient carried a nonsense mutation in *IDH2*, which is also frequently mutated in cancer and has a similar effect to *IDH1*. A third IDH family member, *IDH3A*, which encodes the alpha-subunit of IDH3, carried a non-synonymous mutation (Table S2 in Additional file 1), although its significance is not clear. Further, we found mutations in the tumor suppressor *BAP1* [13] in two patients (Figure 2B). BAP1 is a deubiquitylase associated with protein complexes regulating key cellular pathways, including the cell cycle, cellular differentiation, cell death, gluconeogenesis and the DNA damage response [14]. One patient carried a deletion leading to a frame-shift, along with another non-synonymous mutation. The second patient had a D184V mutation, a position important for catalytic activity, based on sequence similarity to the residue D176 from UCHL1 [15]. While frequent somatic BAP1 mutations have been found in mesothelioma, uveal melanoma and cutaneous melanoma response [14], we report the first finding of BAP1 mutation in HCC. Another notable mutation was a E545K mutation in PIK3CA (Table S2 in Additional file 1), which is a hotspot mutation in the helical domain leading to constitutive activation [16,17]. Several other mutations previously reported in cancer were found as singleton cases (Table S2 in Additional file 1).

Aberrations in the members of the Wnt signaling pathway are reported to be frequent in HCC [6]. Besides point mutations in *CTNNB1* and *AXIN1*, we also found partial deletions in these genes. The *CTNNB1* deletion spans exons 3 and 4 (Figure S3A,B in Additional file 2; Table S5 in Additional file 1). This in-frame deletion removes the amino terminus of β-catenin, a region heavily mutated in multiple cancers (Figure 2C). Since the amino terminus is involved in degradation of β-catenin, the deletion likely results in its stabilization. We previously reported an *AXIN1* deletion [3] that comprises the last three exons of the gene and results in a fusion with *LUC7L* (Table S6 in Additional file 1), likely leading to functional loss.

### Hepatitis B virus integration into the human genome

**Figure 3:**
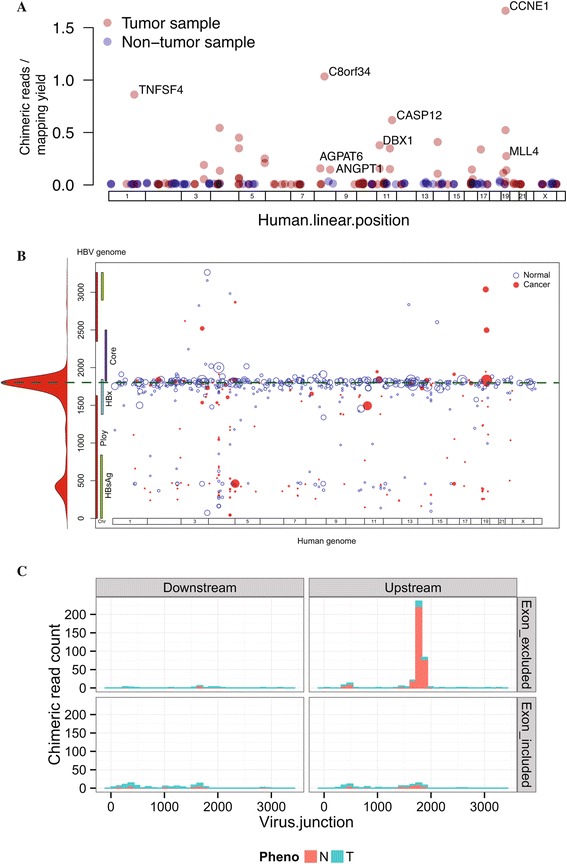
**Hepatitis B virus integration and chimeric transcription in hepatocellular carcinoma. (A)** Viral integrations in 12 whole-genome sequenced samples were determined based on human-viral chimeric reads. Overlapping or nearby (within 500 bp) chimeric reads were clustered together, and the closest gene to the human-viral junction of each cluster was determined. In the plot, each point represents a chimeric read cluster, and the y-axis represents the total number of chimeric reads per cluster per gigabase of mapped human bases for that sample. The human junction is shown on the x-axis. For each sample, the cluster with the highest number of chimeric reads is labeled with the closest gene. **(B)** Two hotspots for viral breakpoints in viral-human chimeric transcripts. Human-viral chimeric RNA reads were clustered based on close vicinity (up to 500 bp). The scatter plot shows the mapping of the clusters on the viral genome (y-axis) versus the linearized human genome (x-axis). The size of the points is proportional to the number of reads belonging to the cluster. The histogram on the y-axis represents the frequency of chimera observed along the viral genome. Two hotspots are observable. The most frequent viral junction is the region at the 3’ end of the *X* gene. A second, less frequent hotspot is seen at the *S* gene. **(C)** Chimeric transcripts show enrichment for viral promoter origin and human exon exclusion. Histograms representing chimeric RNA-Seq read counts are shown. Chimeric reads were classified as downstream, when the viral positive strand was 3’ of the human sequence, or upstream, when the viral positive strand was 5’ of the human sequence (consistent with viral promoter-driven transcription). They were further classified for inclusion or exclusion of human exon sequence. Specific enrichment of the chimeric transcripts can be seen when the viral sequence is upstream of the breakpoint, and human exons are excluded.

HBV integration is another mechanism for influencing gene expression and function in HCC. HBV can randomly integrate into the genome of infected hepatocytes [3,4,18,19]. Recurrent HBV integrations near cancer-related genes like *TERT* [4] indicate that HBV can play a causal role in HCC. From the whole genomes of HBV-infected patients we identified 146 HBV integration sites (Table S3 in Additional file 1), and from their transcriptomes we identified 545 chimeric transcripts (Table S4 in Additional file 1). The number of integration sites per patient ranged from 2 to 28 sites in the tumor samples and 0 to 19 sites in the tumor-adjacent samples. Clonal expansion of HBV-containing hepatocytes was specific to tumors (Figure 3A), as the tumors showed high amounts of human-viral chimeric DNA compared with the tumor-adjacent samples. We found integrations near three known recurrent integration targets [4]: two in the promoter of *TERT*, one upstream of *CCNE1* (Figure S4 in Additional file 2) and another in the third exon of *MLL4* [3]. These integrations occur at high clonal frequency (Table S3 in Additional file 1). The HBV integration near *CCNE1* correlated with extensive genomic instability at this region, featuring at least three inversions and one inter-chromosomal translocation to chromosome 7 (Figure S4 in Additional file 2). Intriguingly, none of the breakpoints disrupted the *CCNE1* coding sequence. Other highly clonal integration sites were mapped near *TNFSF4* and *AGPAT6*, in addition to *ANGPT1* and *CASP12* as previously reported [3].

### Hepatitis B virus-mediated transcription from viral promoters

Besides HBV viral integration at the DNA level, we also performed comprehensive analysis of available RNA-Seq data to identify fusion transcripts between HBV and human sequences. A large number of such chimeric transcripts were identified (Table S4 in Additional file 1). Chimeric transcripts were preferentially fused to two breakpoints in the viral genome: one near the 3’ end of the *X* gene and the other within the *S* gene (Figure 3B; Figure S5A,B in Additional file 2). Human-viral chimeric transcripts may include the viral sequence upstream or downstream of the breakpoint. The former, which is consistent with transcription initiation from a viral promoter, is enriched in the chimera (Figure 3C). If chimeric transcription initiated from a human promoter, we would expect to see enrichment for inclusion of human exons in the chimera; however, that was not the case (Figure 3C). The larger prominence of the breakpoint at the 3’ end of the *X* gene was likely due to linearization (and consequent integration) of the viral DNA at the DR1 site, which was previously identified as a fusion hotspot for human-HBV chimeric RNA [3,18]. Full length HBx has been shown to have oncogenic potential [20]. Interestingly, truncation of HBsAg can confer advantage over immune surveillance [21] and pre-S deletions are associated with development of HCC [22].

### Multiple modes of activation of *TERT*

**Figure 4:**
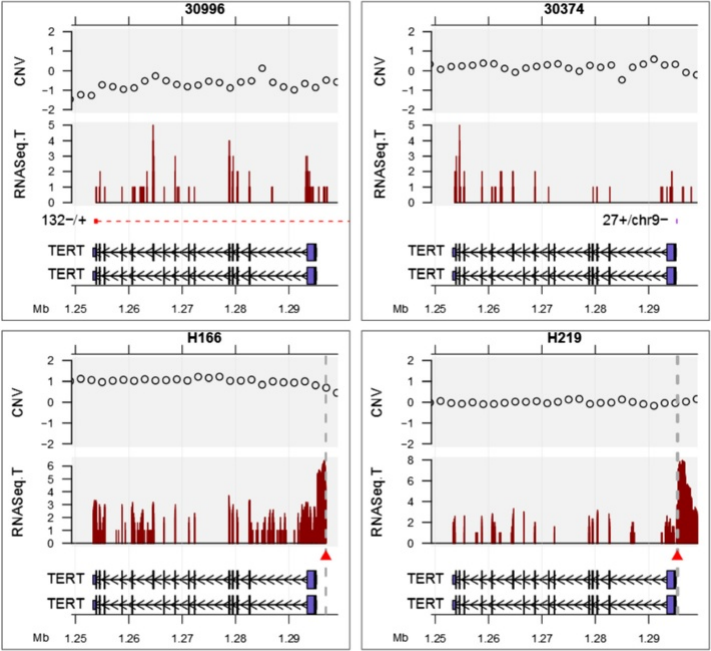
**Multiple modes of** ***TERT*** **activation.** Viral integration and structural variations at the *TERT* locus in tumor samples from four patients. Patient 30996 has an intra-chromosomal inversion supported by 132 whole-genome sequencing reads (red dashed line). Patient 30374 has an inter-chromosomal translocation to chromosome 9 supported by 27 reads (purple block). Viral integration junctions are shown as red triangles and vertical grey lines (patients H166 and H219). RNASeq.T: RNA-Seq coverage in the tumor sample. Coverage is on a linear scale in patients 30996 and 30374, and on a log2 scale in patients H166 and H219. CNV: copy number ratio of the tumor sample relative to the non-tumor sample (log2 scale).

The telomerase gene *TERT* is upregulated in multiple cancers [23]. Here, we observed two modes of perturbation of *TERT*: viral integrations into the promoter of *TERT* in two patients (Figure 4, patients H166 and H219), and translocations in two other patients (Figure 4, patients 30996 and 30374). In all four cases, the breakpoints did not disrupt the exons and *TERT* was expressed in tumor (Figure 4) but not in the matched normal tissue (data not shown). In patient H219, chimeric reads showing fusion between the 5’ UTR of *TERT* mRNA and viral sequence were evident (Table S4 in Additional file 1), suggesting a direct role of HBV in activating *TERT* expression. However, we note that there was bidirectional transcription at this insertion, and transcription in the direction of the *TERT* gene was the least prevalent of the two transcripts resulting from the insertion. In patient H166, evidence for fusion between viral sequence and a region upstream of the *TERT* transcription start site was found, but no direct evidence of a fusion between *TERT* mRNA and viral mRNA was found. In patient 30996, *TERT* was translocated to a region on chromosome 5 that in turn showed extensive rearrangements in a chromothripsis-like fashion (Figure S6 in Additional file 2). Lastly, in patient 30374, the promoter region of *TERT* was involved in an interchromosomal translocation to a region upstream of *RXRA* on chromosome 9 (Table S5 in Additional file 1), likely resulting in misregulation of *TERT. TERT* was not expressed in the non-tumor samples in three out of these four patients, while RNA-Seq data were not available for the fourth patient (30996).

### *LAMA2* is frequently mutated in hepatocellular carcinoma

We found that approximately 14% of the samples analyzed (6/42) had non-silent mutations in *LAMA2* (Figure 1A; Tables S1 and S2 in Additional file 1), a member of the laminin gene family. The relatively high allelic frequencies of these mutations suggest early occurrence during tumor development (Figure 1B). Although the co-occurrence of *LAMA2* mutations and *CTNNB1*/*AXIN1* mutations is low, the apparent exclusivity is not significant (Fisher exact *P*-value = 0.3848). To understand the prevalence of *LAMA2* mutations, we examined data from other studies, and found that approximately 6% (5/88) of the samples in the Asia Cancer Research Group study [4], and approximately 5% (5/104) of the samples in the Riken liver cancer cohort from the International Cancer Genome Consortium also had *LAMA2* mutations. The mutation prevalence will likely vary between different patient cohorts, partly due to extensive heterogeneity observed in HCC. We also found that *LAMA2* is frequently mutated in other cancers, including lung adenocarcinoma (11%), lung squamous cell carcinoma (13%), uterine corpus endometrioid carcinoma (13%), and head and neck squamous cell carcinoma (10%) (data source: The Cancer Genome Atlas).

**Figure 5:**
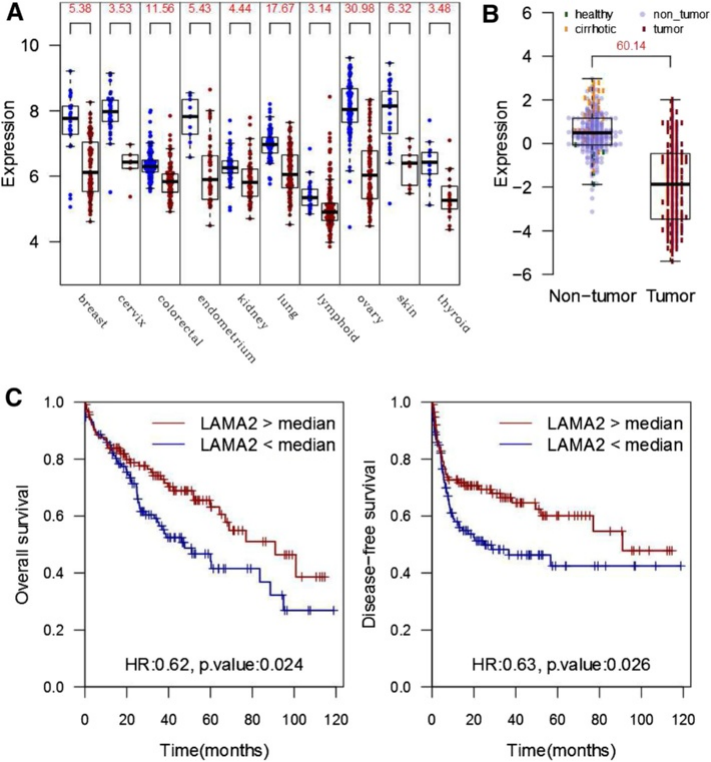
***LAMA2*** **status is associated with clinical outcome in hepatocellular carcinoma. (A)** Microarray-based expression profile of *LAMA2* across a panel of cancer and normal human tissues (Gene Logic, Gaithersburg, MD, USA). Numbers at the top are -log10 *P*-values for a two-sided *t*-test comparing the expression values between the normal (green) and cancer (red) samples of the same tissue. **(B)** Microarray-based expression profile of *LAMA2* in 300 HCC patients from University of Hong Kong (data from Gene Expression Omnibus series GSE25097). The number at the top is the -log10 *P*-value for a two-sided *t*-test comparing the expression values between the non-tumor (blue) and cancer (red) samples. **(C)** Kaplan-Meier curves for survival of *LAMA2*-low versus *LAMA2*-high HCC patients from a cohort of 228 HCC patients from the University of Hong Kong. Patients with low *LAMA2* expression show poorer overall survival (left panel) and disease-free survival (right panel) by log-rank test (*P*-values of 0.024 and 0.026, respectively). HR: hazard ratio between *LAMA*-high and *LAMA*-low samples.

The non-focal nature of *LAMA2* mutations in liver cancer (Figure S7 in Additional file 2) suggests it plays a tumor suppressor role. Accordingly, downregulation of *LAMA2* expression was connected to tumor progression in other tumor types like laryngeal squamous cell carcinoma [24] and breast cancer [25]. We examined a comprehensive panel of tumor tissues for expression profiling, and found downregulation of *LAMA2* across multiple cancer types, most notably in ovarian, lung and colorectal cancer (Figure 5A). In addition, we examined multiple cancer cell lines and found that a decrease in *LAMA2* expression was accompanied by an increase in DNA methylation near the transcription start site (Figures S8 and S9 in Additional file 2). Furthermore, we observed significant downregulation of *LAMA2* expression in a large cohort of un-treated HCC patients with clinical data from the University of Hong Kong [26] (Figure 5B). Promoter DNA methylation, downregulation across multiple cancer types, and mutations in a number of cancer indications support a tumor suppressor role for this gene.

**Figure 6:**
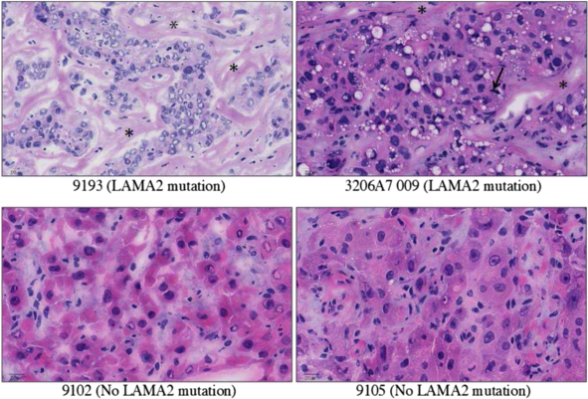
**Morphology of hepatocellular carcinomas with** ***LAMA2*** **mutations.** Hematoxylin and eosin (H&E)-stained sections of HCC with *LAMA2* mutation (upper panels, 9193, 3206A7 009) show poorly differentiated neoplastic hepatocytes with cellular and nuclear pleomorphism, mitotic figures (arrow) and moderate to abundant stroma (asterisks). H&E-stained sections of HCC with wild-type *LAMA2* generally have more uniform, well-differentiated neoplastic cell morphology (lower panels). Scale bar is 20 μm.

To test whether laminin deficiency could impact the clinical outcome of HCC patients, we examined the patient survival data from the University of Hong Kong cohort of HCC patients (228 HCC samples with survival data). We found that patients with lower *LAMA2* expression level showed significantly worse survival outcome (Figure 5C; *P*-value = 0.024, log-rank test). The effect size was greater upon stratifying the patients into upper and lower quartiles instead of median-based stratification (Figure S10 in Additional file 2). Among the 5 *LAMA* family members, only *LAMA2* showed a significant relation with clinical outcome. Moreover, patients with low *LAMA2* expression were 30% more likely to have tumor recurrence (odds ratio = 1.7, *P* = 0.034, Chi-Square test). Thus, *LAMA2* low expressing tumors appear to represent a more aggressive subtype of HCC. Interestingly, compared with tumors with wild-type *LAMA2*, tumors with *LAMA2* mutations showed histopathological features of poorly differentiated tumors, with substantial cellular and nuclear atypia and moderate to abundant stroma interspersed between cells (Figure 6). To understand the underlying molecular mechanism of such differences in clinical outcome, we compared the *LAMA2*-low with the *LAMA2*-high tumors and found striking enrichment for upregulation of cell cycle genes (Figure S11 in Additional file 2). A similar trend was also observed in breast, colorectal and lung cancers (Figure S11 in Additional file 2). Therefore, the *LAMA2*-deficient samples represent a subgroup of highly recurrent and proliferative hepatocellular carcinomas, and *LAMA2*-based stratification appears to apply to other cancer types as well.

## Discussion

Multiple efforts using deep sequencing of HCC are now revealing important players in this heterogeneous disease. Previous genomic studies have implicated the role of tumor suppressor *TP53*, Wnt-signaling components *CTNNB1* and *AXIN1*, telomerase *TERT*, cell cycle regulator *CCNE1*, and chromatin regulators like *MLL4*, *ARID1A* and *ARID2*, although different studies have identified different subsets of these players. Here we report low frequency mutations in tumor suppressor *BAP1* and metabolic genes *IDH1* and *IDH2* in HCC. Although mutations in these genes have been reported in other cancer types such as glioblastoma and mesothelioma [8,14], *BAP1*, *IDH1* and *IDH2* have not been previously linked to HCC. Since mutant IDH1 has been pursued as an anticancer target in glioma [27], it is conceivable that HCC patients carrying the R132 mutation can also benefit from such IDH1 inhibitors. Similarly, HCC patients with PIK3CA E545K mutation can benefit from inhibitors effective against the kinase activity of this mutant [28]. It is conceivable that other *PIK3CA* mutations may also be present in HCC.

Interestingly, we observed frequent mutations in the extracellular matrix gene *LAMA2* in liver cancer patients. Specific upregulation of *LAMA2* expression in cirrhotic hepatocytes (Figure 5B) suggests that basal laminae may be required for controlled regeneration following liver injury. Low expression of *LAMA2* is tied to poor survival outcome, high recurrence of HCC, and upregulation of cell cycle genes. We compared the *LAMA2* expression profile, using the transcriptome data from 12 tumors, with those of poor-prognosis stemness markers like *EPCAM*, *PROM1* (CD133), *THY1* (CD90), *NCAM* and *KRT9* (CK19), but did not find any conclusive evidence of correlation with these markers, although there was a weak correlation with CD90 (Pearson correlation coefficient = 0.65). It is conceivable that functional LAMA2 in the extracellular matrix may keep the proliferation of regenerating hepatocytes in check, and defective or lack of LAMA2 facilitates tumor progression. This is also supported by the fact that the loss of cell surface anchoring to basal laminae has been found to promote tumor growth and cell proliferation [7]. Moreover, soluble laminin in culture can suppress cell proliferation in mammary epithelial cells [29]. DNA methylation at the *LAMA2* promoter region found by us and others [30] suggests that epigenetic mechanisms may target *LAMA2* in multiple cancers. These findings suggest the importance of the extracellular matrix during HCC development and perhaps other types of cancers as well. This has potential implications on stratification of HCC patients and on decisions about therapeutic options for such categories of patients.

While the expression of *LAMA2* seems to be related to DNA methylation, the *TERT* gene appears to be influenced by other modes of genomic alteration. In two of our HCC samples, the HBV viral DNA is integrated into the promoter of *TERT*, leading to the activation of *TERT* expression. In two other HCC patients, viral-independent translocation was observed, juxtaposing the *TERT* promoter on other active genomic regions. All four such patients exhibit much elevated *TERT* gene expression compared with adjacent liver tissues based on our RNA-Seq data (Figure 4). More recently, point mutations have been found in the promoter region of *TERT* in multiple types of cancer, leading to higher *TERT* mRNA expression [31,32]. Clearly, *TERT* activation is common in human cancers and is likely oncogenic, but the mechanism of gene activation may differ between cancer types. While we did not find any point mutations in the *TERT* promoter in HCC, other HBV-dependent and HBV-independent genomic events seem to be involved in alternative mechanisms of *TERT* activation.

**Figure 7:**
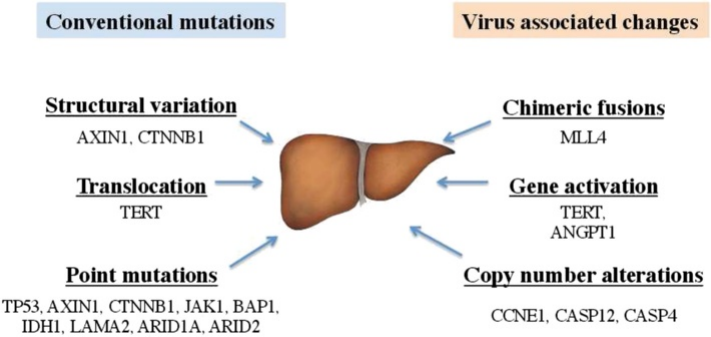
**Diverse modes of genetic alterations in hepatocellular carcinoma.** Conventional mutations are the changes that can also be seen in other cancers, while the virus-associated changes are unique to HBV-infected HCC tumors. Examples of genes associated with each genetic alteration are shown, compiled from multiple studies.

Overall, there is a constellation of genomic alterations in HCC (Figure 7). Among conventional mutations, point mutations are clearly the most studied type of changes in HCC due to mature technologies for detecting such mutations. Large coding region deletions, such as the *CTNNB1* deletion we report here (Figure 2C), are usually understudied but their functional importance should not be ignored. Perhaps the most notable genomic alterations in the HBV-positive patients are various changes caused by viral DNA integration into the human genome. Based on our work and that of others [3–5], patterns of HBV-based mutagenesis are starting to emerge. In some patients, integrated HBV viral promoter would activate nearby genes such as *TERT* and *ANGPT1*. In others, HBV integration sites occur in the middle of human genes such as *MLL4*, causing gene disruption and/or viral-human chimera transcripts. HBV integration also leads to local genomic instability, further causing DNA copy number changes [3]. Such diverse modes of genomic alteration add genetic complexity to HCC (Figure 7), likely leading to its highly heterogeneous nature. Deeper understanding of these complex patterns of mutations could also provide better understanding of the etiology of HCC and possibly lead to development of novel anti-HCC therapeutics. For example, the HBx-MLL4 chimera fusions potentially produce cancer-specific proteins that can be specifically targeted therapeutically, and the *TERT* gene activation modes may suggest different diagnostic markers for a subset of HCC patients. With more comprehensive genomic characterization of liver cancer patients, more detailed and reproducible cancer subtypes will emerge that can eventually guide clinical diagnostics and treatment management.

## Conclusion

We found diverse modes of genomic alteration in HCC that affect important players in this disease. Point mutations and structural variations affected both *AXIN1* and *CTNNB1*. We found HBV integrations in the *TERT* promoter, and virus-independent *TERT* translocations, likely leading to activation of *TERT* expression. HBV integrations occurred in *MLL4*, *CCNE1* and *TERT*, leading to increased expression of *CCNE1* and *TERT*, or HBx-MLL4 fusion. *TP53*, *AXIN1* and *CTNNB1* were the most frequently mutated genes in our cohort. *LAMA2* showed high allelic frequency mutations, and we also found point mutations in *BAP1*, *IDH1*, *IDH2* and *PIK3CA*.

## Materials and methods

### Samples and sequencing

Frozen tissue and peripheral blood mononuclear cell (PBMC) samples were obtained from commercial sources (Seracare LifeSciences, Milford, MA, USA; ProteoGenex, Culver City, CA, USA and Indivumed, Baltimore, MD, USA). Appropriate informed consent and institutional review board approval was obtained based on a representation and warranty from the vendors. Four-micron thick frozen sections were obtained from both primary HCC and the matched non-neoplastic liver tissue for histopathological evaluation by standard hematoxylin and eosin stain. Two distinct sets of samples were used for whole-genome sequencing and exome-sequencing. For whole-genome sequencing, tumor and tumor-adjacent samples from 12 patients were sequenced at 78× to 132× coverage using the Complete Genomics (Mountain View, CA, USA) platform. Of these 12 patients, 4 were reported previously [3]. Eight patients were confirmed to be HBV-positive by PCR. Thirty sets of tumor and tumor-adjacent samples were exome-sequenced using Illumina Hi-Seq. Exome sequencing was performed at a median read count of 79.9 million reads (range was 44.2 to 250 million). Uniquely mapped reads (35 to 213 million; 67.5 million median value) were used for variant calling.

### Variant calling

High-throughput reads from whole-genome sequencing were mapped by Complete Genomics to the human genome (NCBI build 37). The mean mate gap post-mapping was approximately 311 bp. Single nucleotide variation, short indels and substitutions, large-scale structural variation, and copy number variation data were provided by Complete Genomics (standard pipeline version 2.0.1.6). Somatic mutations were called using open-source cgatools v.1.5 [33]. Mutations with a somatic score greater than -5 were retained. Further filtering of somatic mutations was done by removing mutations that were common with dbSNP build 132, a set of 69 normal genomes from Complete Genomics [34], the 1000 Genomes Project, and 2,440 exomes from NHLBI [35]. Any mutations that were also present in COSMIC v.62 were retained. The resulting set of mutations constitutes high-confidence mutations used in all the analyses in this study. For determining viral reads, whole-genome sequencing reads where at least one arm was not mappable to the human genome were mapped to a consensus HBV genome sequence using mapping tools from Real Time Genomics® (Hamilton, New Zealand).

Exome sequencing reads from the Illumina platform were mapped to UCSC human genome (GRCh37/hg19) using the default parameters of the BWA software [36]. Duplicate read removal and local realignment were performed as described previously [37]. Variant calling was performed on the tumor and normal BAM files using the Strelka algorithm [38] with the BWA default configuration file and a minimum variant quality of 30. Known germline variations represented in dbSNP build 131 [39] or 6,515 previously published normal exomes [40], but not represented in COSMIC v62 [41], were excluded. In addition, predicted somatic variations were additionally filtered to have a minimum variant allele frequency of 5% and not exceeding that number in the matched normal sample.

Somatic mutation calls from the whole genomes and exomes were pooled and the number of tumor samples carrying a mutation was tallied for each gene. For genes mutated in three or more samples, we selected those that were preferentially mutated at high allele frequencies across multiple patients. This eliminated some genes that were apparently mutated in a large number of tumors, but showed low allelic frequencies across these tumors (Figure 1B). Among the genes that were mutated in two samples, we focused on cancer gene census genes. Among these cases, *IDH1* was recurrently mutated at position R132, and *BAP1* carried three high allelic frequency mutations, two of them in the same tumor. Other notable mutations included a nonsense mutation in IDH2 at amino acid 63, and a E545K mutation in PIK3CA, which is a hotspot mutation in several cancers [16].

### Mapping to the hepatitis B virus genome

Complete Genomics reads were mapped to the HBV consensus genome using the Real Time Genomics® software [42]. The subset of reads with minimum one mate pair mapping to the HBV genome were mapped to a ‘hybrid genome’ - a combination of the human genome (hg19) and the HBV virus genome. The hybrid genome was created by merging the FASTA files of the human genome (hg19) and the HBV consensus genome, so that the viral genome is presented as an additional ‘pseudo’ chromosome of the human genome. Based on a Phred-like quality score cutoff of minimum 20, aligned reads were further filtered for reads with one mate pair mapping uniquely to the human genome and the other mate pair mapping uniquely to the HBV genome.

### Determination of viral integration sites and viral-human fusion transcripts

For both whole-genome sequencing and transcriptome sequencing, human-viral chimeric reads were grouped into chimeric clusters. If a boundary of a read was within 500 bp of the boundary of another read on the human as well as the viral arm, the two reads were assigned to the same cluster. Each cluster represents a human-viral junction, and two such junctions are expected per viral insertion site in the human genome. However, two junctions were not always detectable, likely due to lack of coverage or difficulty in mapping at one of the junctions. For whole-genome sequencing, we identified 146 such clusters (Table S3 in Additional file 1), and 612 clusters for RNA-Seq (Table S4 in Additional file 1), with at least two reads per cluster. Since two nearby chimeric clusters on the human genome may represent the same viral integration event, for the purpose of estimating the clonality of each integration (Figure 3A), we reduced this redundancy by retaining only the cluster with the higher number of chimeric reads, if the boundaries of two clusters are within 2 kb of each other on the human genome.

Chimeric transcripts showed two hotspots for breakpoints on the viral genome (Figure 3B). To examine these in more detail, we classified the human-viral breakpoints from whole genome and transcriptome data into two types: one where the fused viral sequence is upstream of the breakpoint in the viral genome, and the other where the viral sequence is downstream of the breakpoint (Figure 3C). The enrichment observed was especially striking for chimeric transcripts with upstream viral breakpoints, indicating that viral promoters (for *X* and *S* genes) are the likely driving factors for these fusion transcripts.

### *LAMA2* expression in multiple tissues

Expression data were obtained from a panel of 37 tissues from Gene Logic (Gaithersburg, MD, USA; Affymetrix HG-U133 platform, representing 3,600 normal and 1,701 neoplastic samples from different human tissues). Tissues that showed significant expression change in *LAMA2* (*P* ≤ 0.001, two-sided *t*-test) are shown (Figure 5A). Expression was significantly decreased in breast, cervix, colorectal, endometrium, kidney, lung, lymphoid, ovary, skin, and thyroid tumor tissues.

### DNA methylation analysis

DNA methylation was measured using Illumina Infinium 450 K BeadChip and preprocessed using the Bioconductor lumi package [43] with default settings (within-sample quantile color bias adjustment plus across-sample quantile normalization of pooled probe intensities). The methylation plot was produced using Bioconductor methyAnalysis package.

### Relation between clinical data and *LAMA2* expression

A cohort of 228 primary HCC samples from the University of Hong Kong was used for evaluating the effect of *LAMA2* expression on survival. Statistical analysis was performed using IBM SPSS version 16.0 (Armonk, NY, USA) for Windows. For clinical correlation analysis with survival rates, continuous variables were modeled as categorical variables. Univariate analysis of *LAMA2* expression on overall survival and disease-free survival rates was performed using the Kaplan-Meier method. The subjects were equally divided into high- and low-expression arms using the median value as cutoff (Figure 5C). To achieve more stringent stratification, upper and lower quartiles were used instead of median-based separation. The resulting Kaplan-Meier plots show a higher level of separation (Figure S10 in Additional file 2).

### Detection of structural variation and potential gene fusion events

We utilized the paired-end nature of the sequencing reads to detect structural variations (Table S5 in Additional file 1) and potential gene fusion events (Table S6 in Additional file 1). The method used to detect structural variation was as previously described [3]. We further filtered somatic structural variants for matched or unrelated normal samples. For these putative somatic structural variants, we further searched for structural variation events that potentially lead to fusion gene events by the following criteria: 1) the somatic structural variant breakpoints defined by DNA-Seq overlap with a pair of distinct human genes (refGene); 2) the RNA-Seq data support the fusion occurred between these two fusion partners. For detecting fusion at the RNA level, we applied both ChimeraScan [44] and gStruct (Thomas Wu, unpublished). The somatic structural variants that lead to putative fusion events are reported in Table S5 in Additional file 1.

### Data availability

Sequence data have been deposited at the European Genome-phenome Archive [45], which is hosted by the EBI, under accession number EGAS00001000824.

ICGC mutation data were accessed from the ICGC data portal [46]. The raw data from this study can be obtained from the European Genome-phenome Archive, study accession EGAS00001000678. Expression data for *LAMA2* from the university of Hong Kong are available from the Gene Expression Omnibus repository, with accession GSE25097. *LAMA2* mutation data from Asia Cancer Research Group study can be accessed at [47]. The Cancer Genome Atlas data were obtained from dbGaP (study accession phs000178.v8.p7).

## Additional files

Additional file 1: Table S1. High confidence mutations in 12 HCC tumors from whole-genome sequencing. **Table S2.** High confidence mutations in 30 HCC samples from exome sequencing. **Table S3.** Viral integrations identified from whole-genome sequencing of eight HCC patients carrying HBV infection. **Table S4.** Summary of human-viral chimeric RNA reads identified from RNA sequencing of eight HCC patients carrying HBV infection. **Table S5.** Somatic structural variation breakpoints identified computationally from whole-genome sequencing of 12 HCC patients. **Table S6.** Somatic fusion transcripts identified computationally from RNA sequencing and whole genome sequencing of 12 HCC patients. Additional file 2: Figure S1. Somatic mutation frequency and mutation signature from whole genome sequencing of 12 HCC patients. **Figure S2.** Exome-based somatic mutation frequency in 30 HCC patients. **Figure S3.** *CTNNB1* structural variant in patient 30147. **Figure S4.** Structural variation and viral insertion near *CCNE1*. **Figure S5.** Frequencies of human-viral chimeric reads based on whole genome and transcriptome. **Figure S6.** Translocation of *TERT* in patient 30996. **Figure S7.** Non-silent *LAMA2* mutations. **Figure S8.** DNA methylation and expression of *LAMA2* in breast cancer cell lines. **Figure S9.** DNA methylation and expression of *LAMA2* in lung cancer cell lines. **Figure S10.** Survival analysis for *LAMA2*-low and *LAMA2*-high patients. **Figure S11.** *LAMA2* downregulation is associated with cell cycle regulation..

## Abbreviations

bp: base pair
HBV: hepatitis B virus
HCC: hepatocellular carcinoma
PCR: polymerase chain reaction
UTR: untranslated region
