# Supplementary material for: Diverse modes of genomic alteration in hepatocellular carcinoma
Source: Genome Biol. 2014 Aug 26;15(8):436. doi: 10.1186/s13059-014-0436-9 (PMC4189592; doi:10.1186/s13059-014-0436-9)
Supplement: Additional file 2: Figure S1. — Somatic mutation frequency and mutation signature from whole genome sequencing of 12 HCC patients. Figure S2. Exome-based somatic mutation frequency in 30 HCC patients. Figure S3. CTNNB1 structural variant in patient 30147. Figure S4. Structural variation and viral insertion near CCNE1. Figure S5. Frequencies of human-viral chimeric reads based on whole genome and transcriptome. Figure S6. Translocation of TERT in patient 30996. Figure S7. Non-silent LAMA2 mutations. Figure S8. DNA methylation and expression of LAMA2 in breast cancer cell lines. Figure S9. DNA methylation and expression of LAMA2 in lung cancer cell lines. Figure S10. Survival analysis for LAMA2-low and LAMA2-high patients. Figure S11. LAMA2 downregulation is associated with cell cycle regulation.. [file 13059_2014_436_MOESM2_ESM.pdf]

A

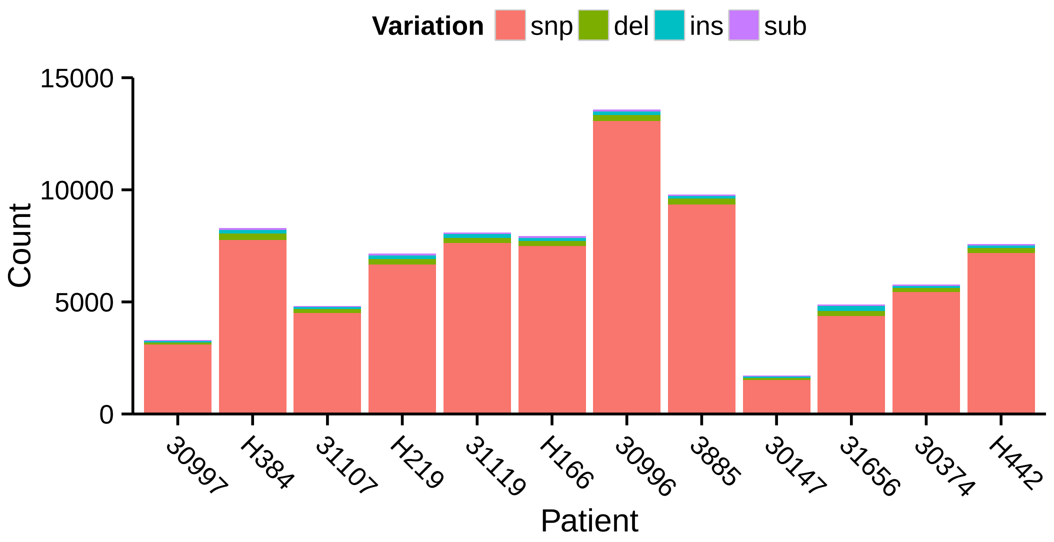

B

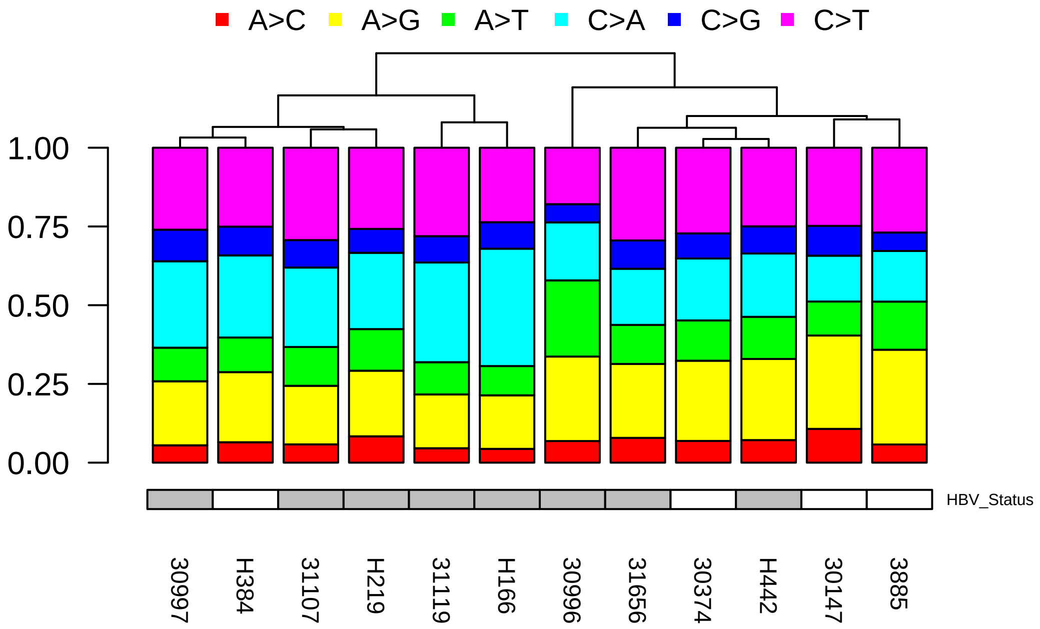

Figure S1. **A**. Number of high confidence somatic mutations, short indels and multi-base substitutions is shown for 12 whole-genome sequences. **B**. Fractions of high confidence somatic mutations (all 6 types of single base changes) per patient. Samples were hierarchically clustered based on the fractions of high-confidence, somatic single-base substitutions. Clustering was done using the Euclidean distance metric and Ward clustering. Only the whole-genome samples were used for this analysis. HBV infection status is shown in grey.

Supplementary Figure 1. Jhunjhunwala et al.

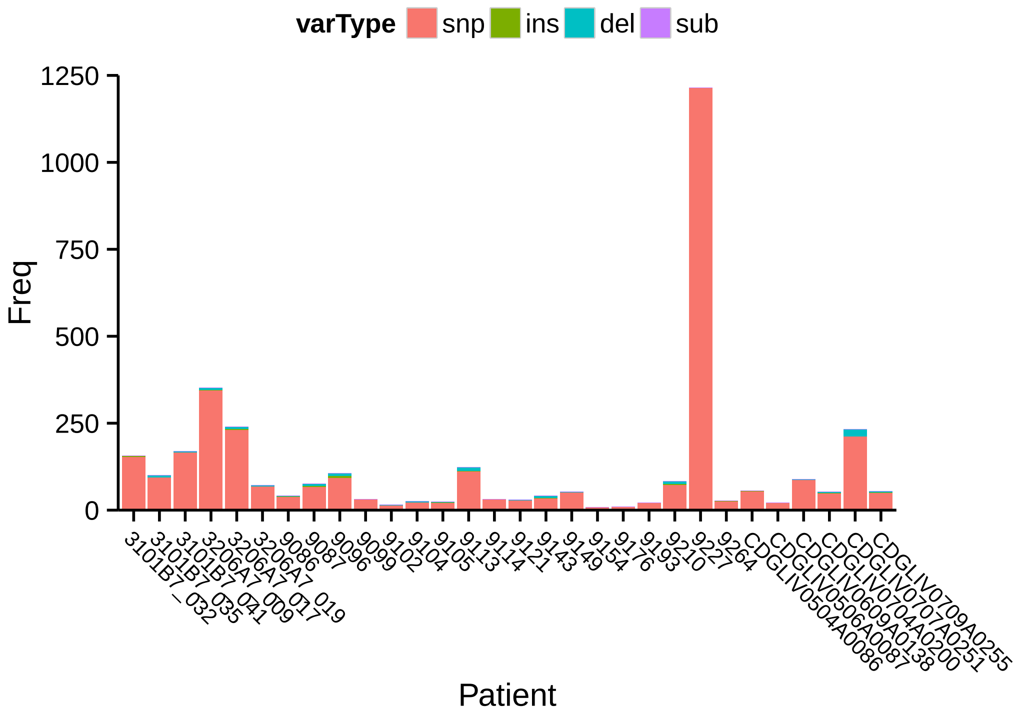

Figure S2. Number of high confidence somatic mutations, short indels and multi-base substitutions is shown for 30 exomes. Patient 9227 is hyper-mutated.

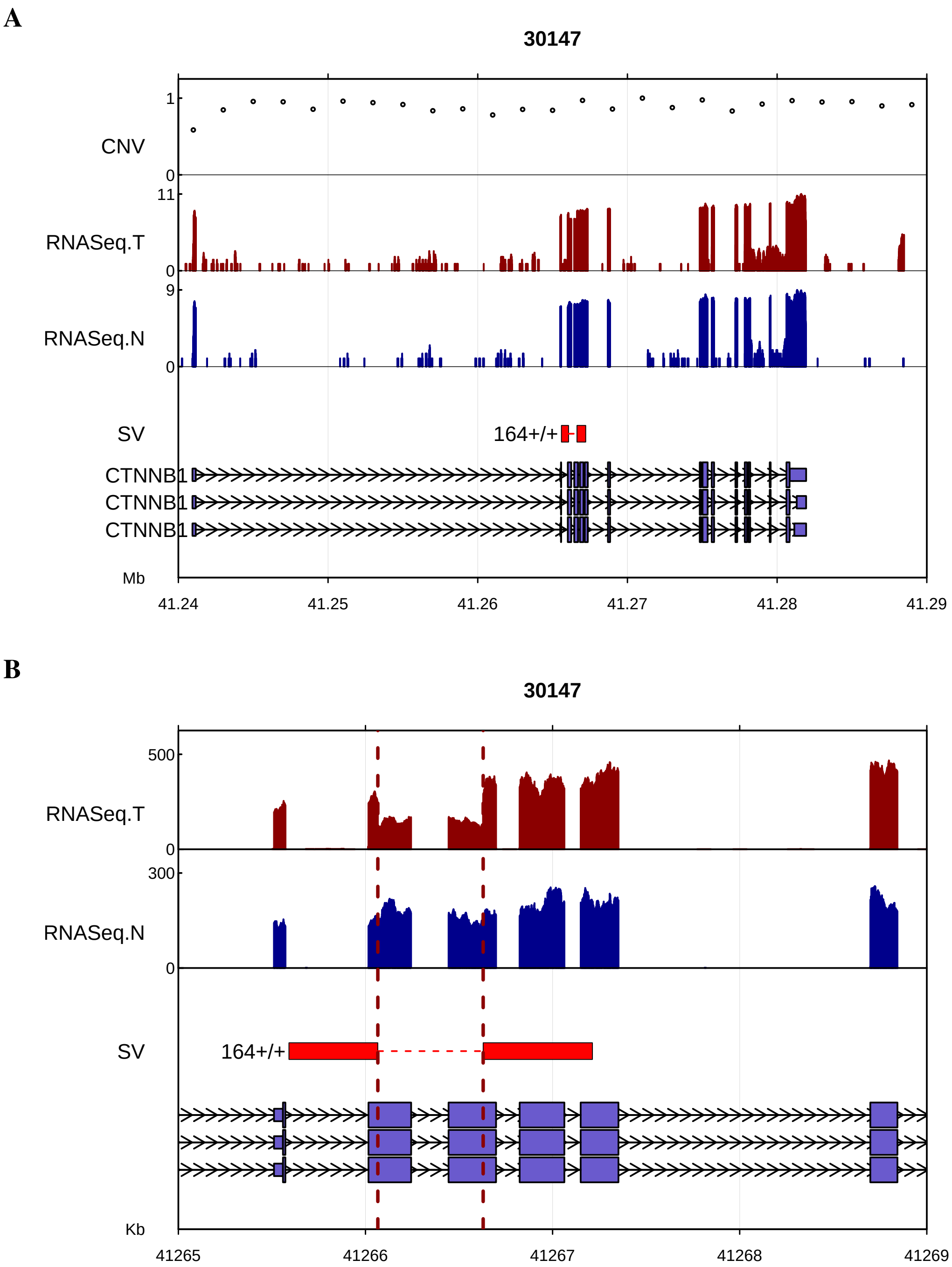

Figure S3. **A**. Summary of whole-genome and transcriptome characteristics at the *CTNNB1* structural variation observed in patient 30147. SV: Structural variation. RNASeq.N: RNA-seq coverage in the non-tumor sample. RNASeq.T: RNA-Seq coverage in the tumor sample. RNA-Seq coverage is shown on log2-scale. CNV: Relative copy number of the tumor sample compared with the non-tumor sample. The red boxes in the SV track show the cluster of 164 reads from whole-genome sequencing which provide evidence for a 563 bp deletion spanning the 3rd and 4th exon of *CTNNB1*. RNA-Seq coverage in the tumor sample over this deleted region is reduced to half the coverage of the rest of the exons 3 and 4 in the tumor sample, but not in the non-tumor sample. **B**. Detailed view of the deleted region of *CTNNB1*. RNA coverage is shown on absolute scale instead of log2-scale. Tumor-specific reduction in coverage is observable at the deletion region. Coverage is reduced to approximately half of the coverage at the region immediately adjacent to the deletion.

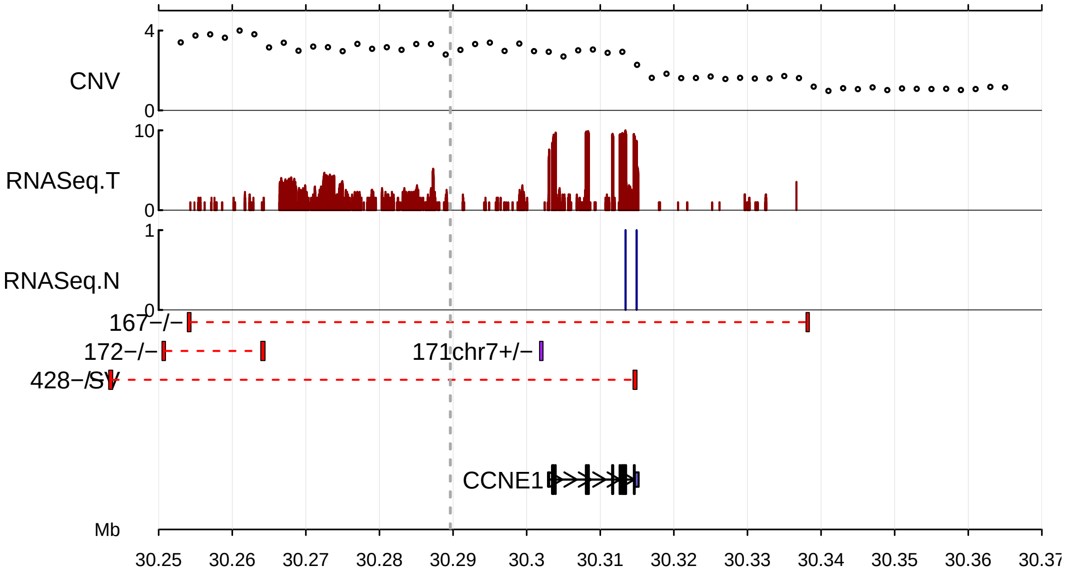

Figure S4. Summary of copy number variation, structural variation and transcription at the *CCNE1* viral integration observed in patient H219. SV: Structural variation. RNASeq.N: RNA-seq coverage in the non-tumor sample. RNASeq.T: RNA-Seq coverage in the tumor sample. CNV: Copy number of the tumor sample relative to the non-tumor sample. Viral integration is shown as a vertical, dashed gray line. Three separate chromosomal breaks are seen as evidenced by three clusters of reads suggesting structural variation. Varying levels of copy number variation are also observable. Transcription is observable in the tumor sample, but not in the non-tumor sample.

A

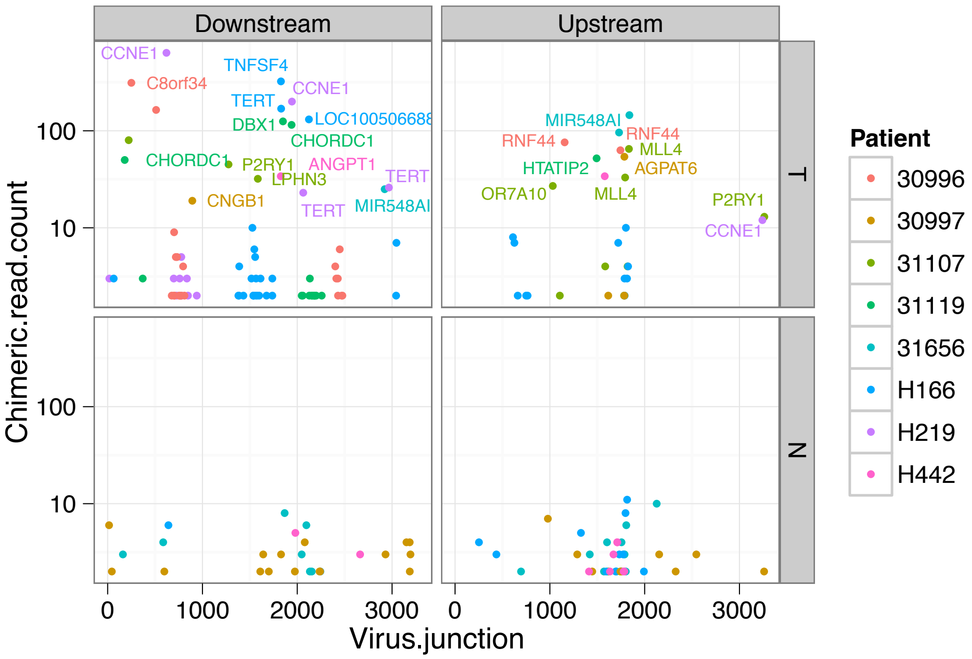

B

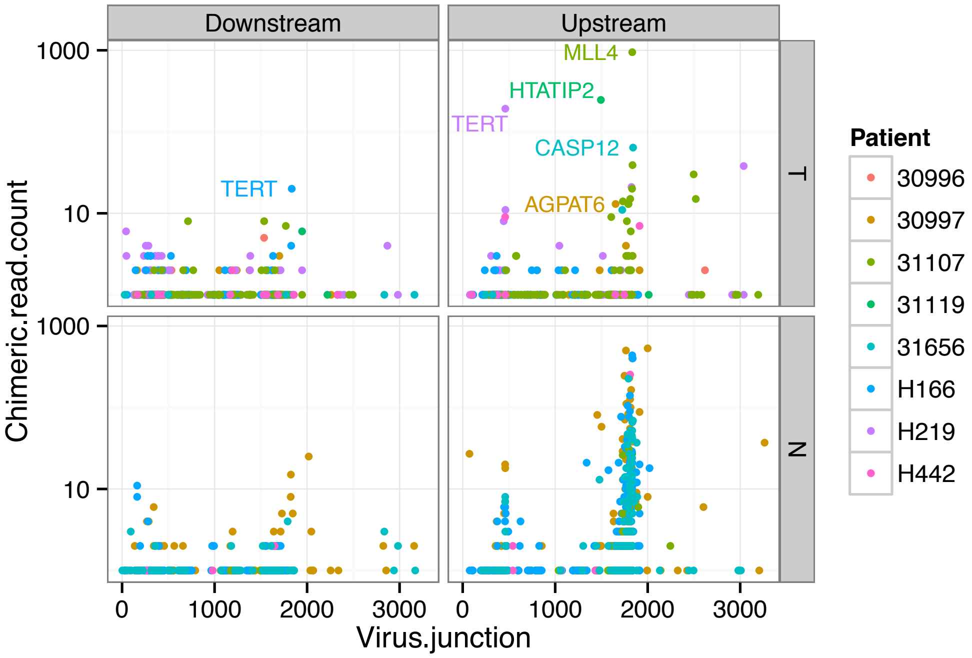

Figure S5. **A**. Human-viral chimeric DNA from the tumor (T) or tumor-adjacent tissue (N) from 12 whole-genome sequencing samples was classified into two categories. The upstream category includes chimera where the viral sequence included in the chimera was upstream of the viral breakpoint. Chimera containing viral sequence downstream of the viral breakpoint were classified as downstream. The total chimeric read count for each cluster is shown on the y-axis, while the x-axis represents the viral junction. **B**. Human-viral chimeric RNA clusters from the tumor (T) or tumor-adjacent tissue (N) from 12 patients. The samples are classified in the same manner as above.

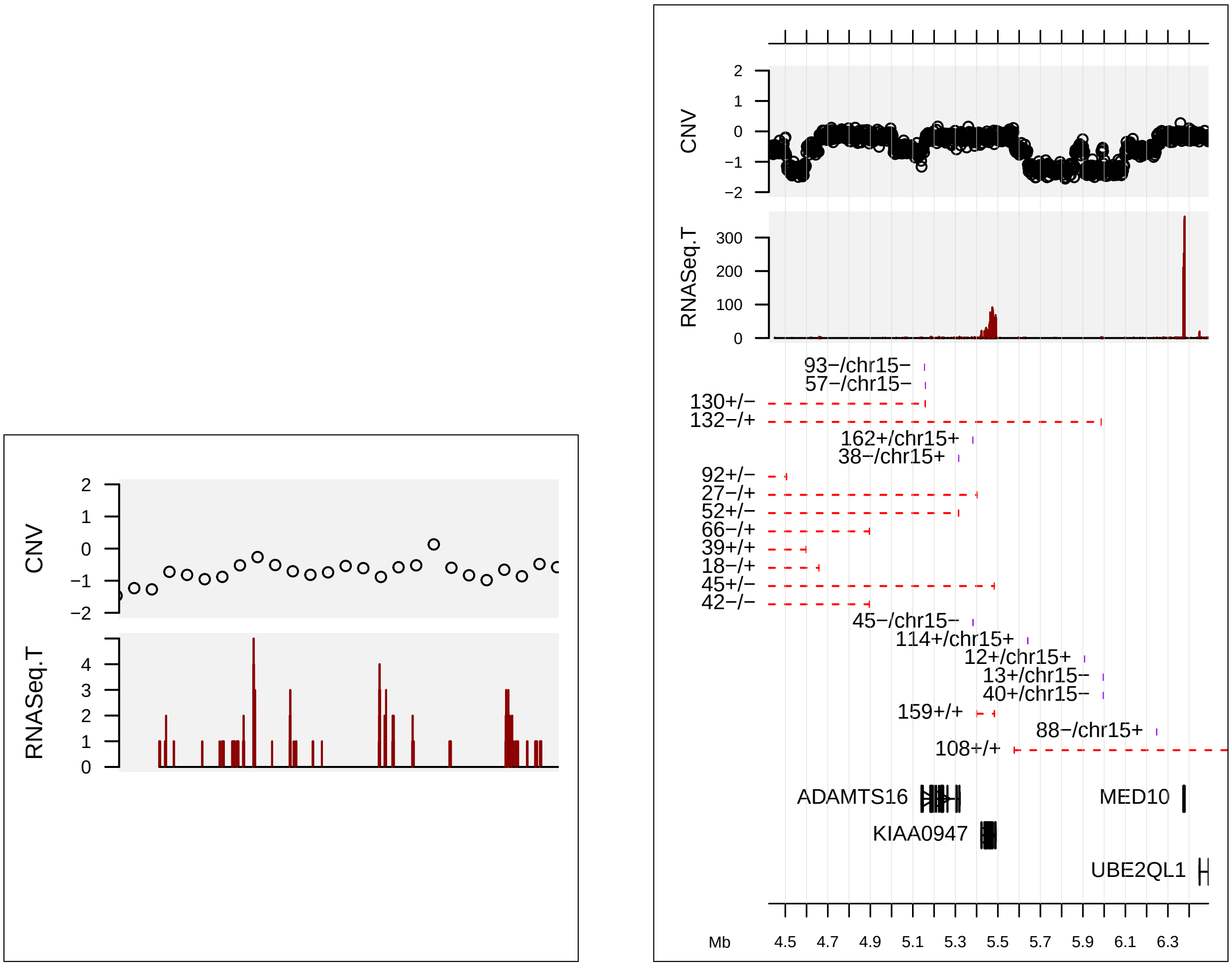

Figure S6. Translocation of *TERT* in patient 30996. An intra-chromosomal translocation to a region 5Mb downstream on chromosome 5 was supported by 132 reads. This region featured several other intra-chromosomal translocation events, and inter-chromosomal translocation events to chr 15 (Right panel).

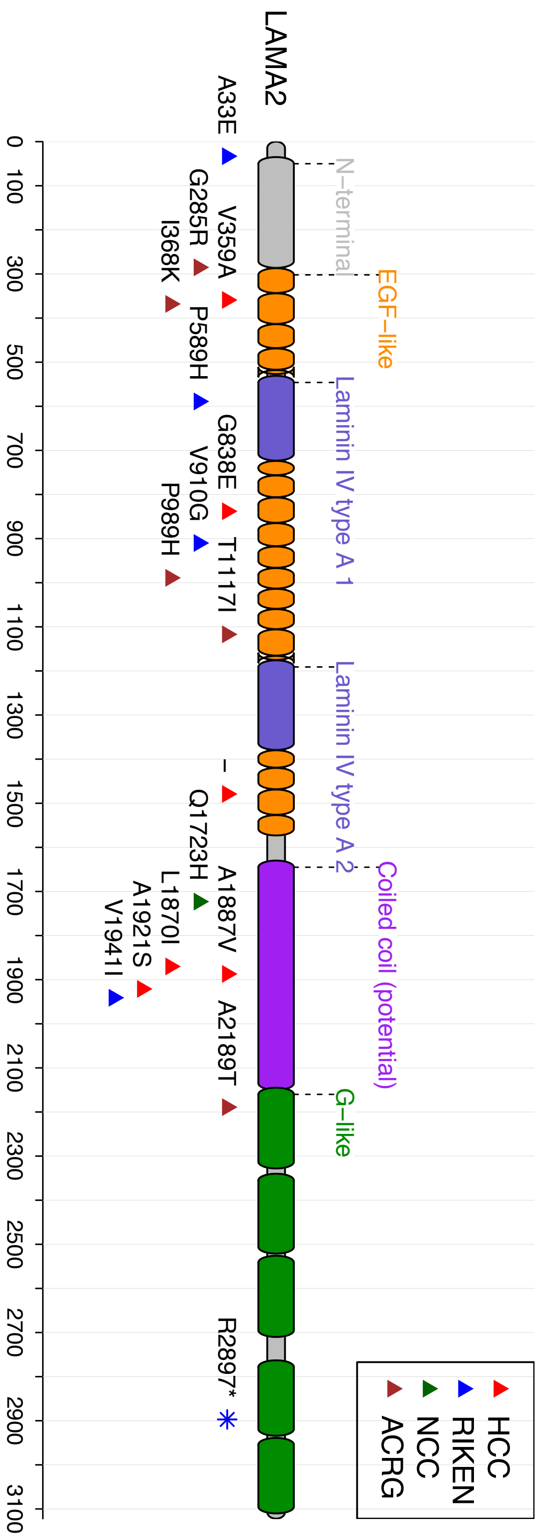

Figure S7. Non-silent mutations in *LAMA2* from the current study (HCC), two ICGC cohorts (NCC and RIKEN), and ACRG.

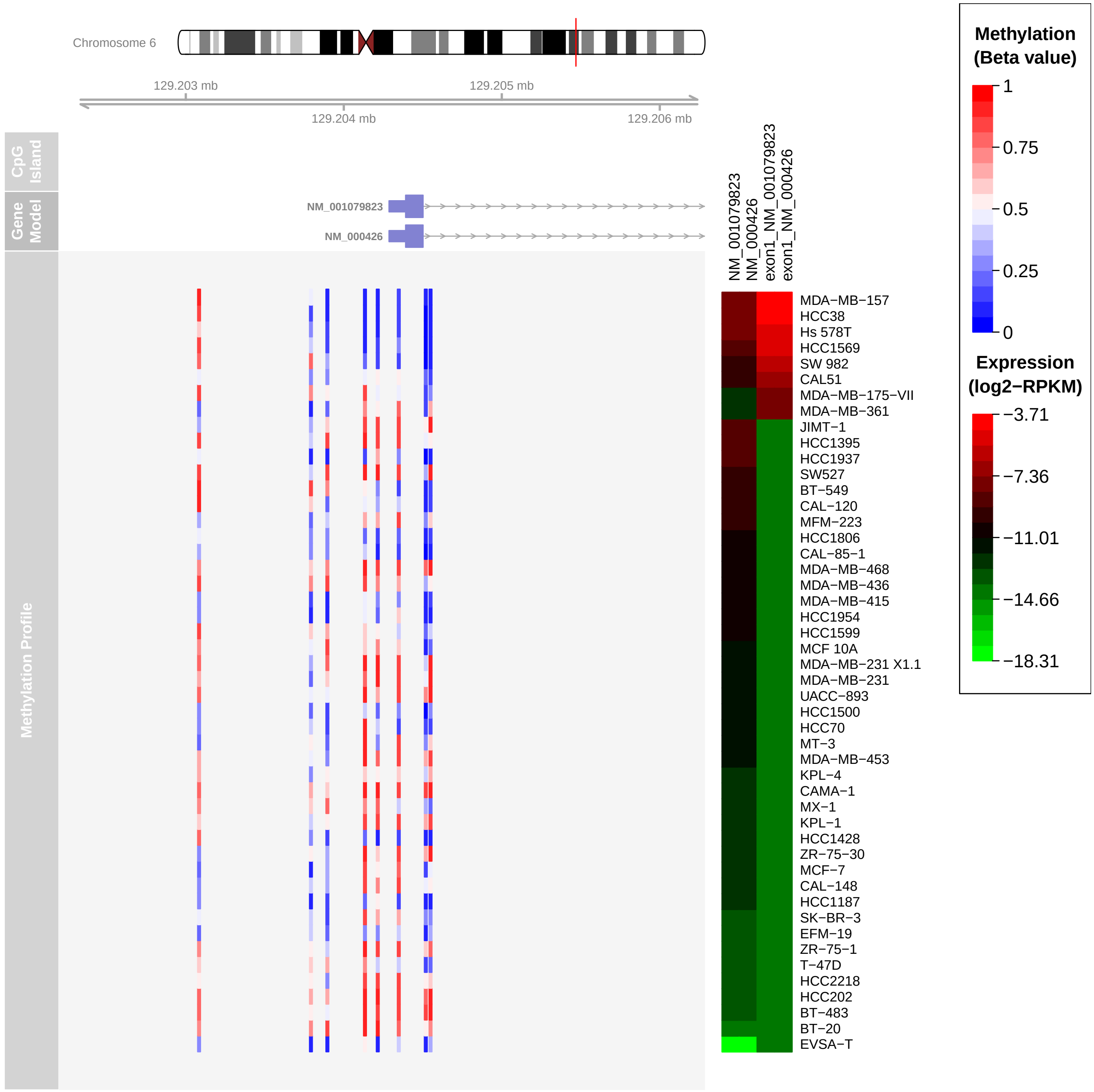

Figure S8. DNA methylation and expression of *LAMA2* in breast cancer cell lines. Multiple DNA methylation probes are shown near the transcription start site. Expression level of *LAMA2* is shown for the whole transcript, and for the first exon alone.

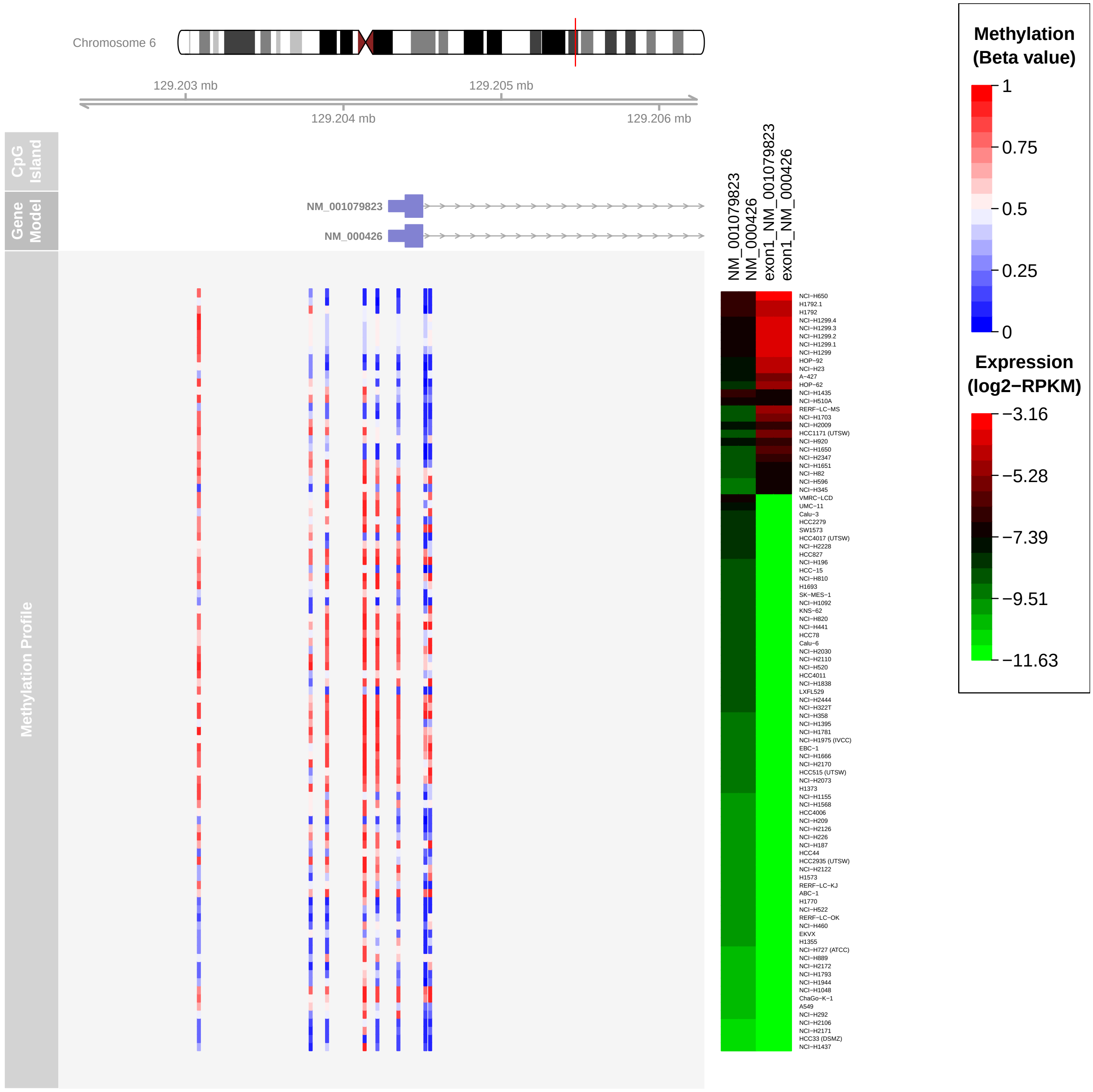

Figure S9. DNA methylation and expression of *LAMA2* in lung cancer cell lines. Multiple DNA methylation probes are shown near the transcription start site. Expression level of *LAMA2* is shown for the whole transcript, and for the first exon alone. Expression is shown for two different isoforms of *LAMA2*.

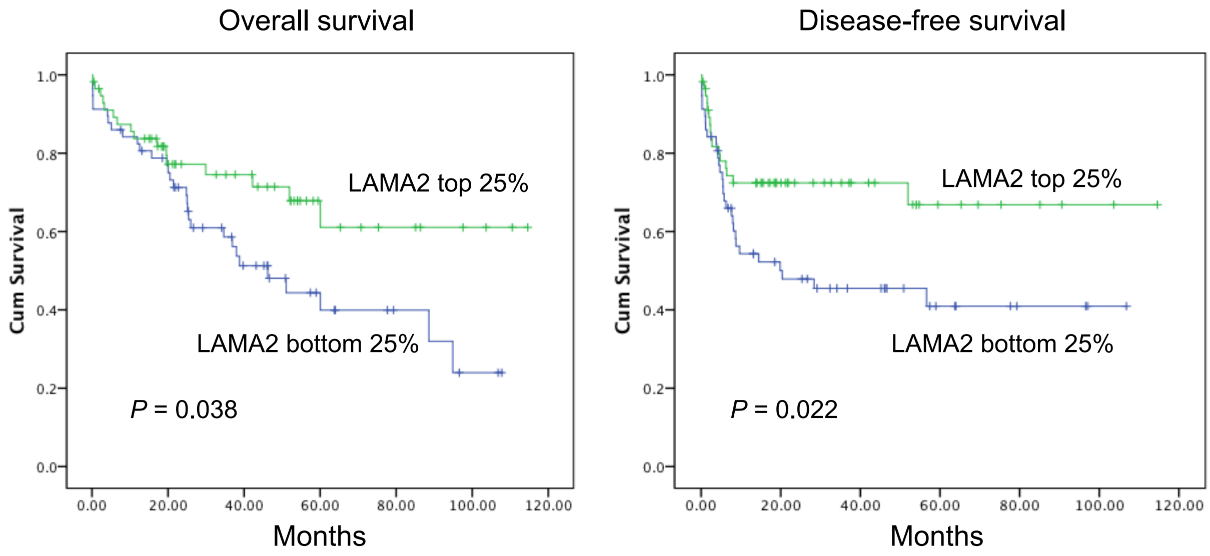

Figure S10. Kaplan-Meier curves comparison of survival between *LAMA2*-low and *LAMA2*-high patients in a cohort of 228 primary HCC specimens from University of Hong Kong. Patients with low *LAMA2* expression show poorer overall survival and disease-free survival by log-rank test. Patients were stratified into top and bottom quartiles based on *LAMA2* expression.

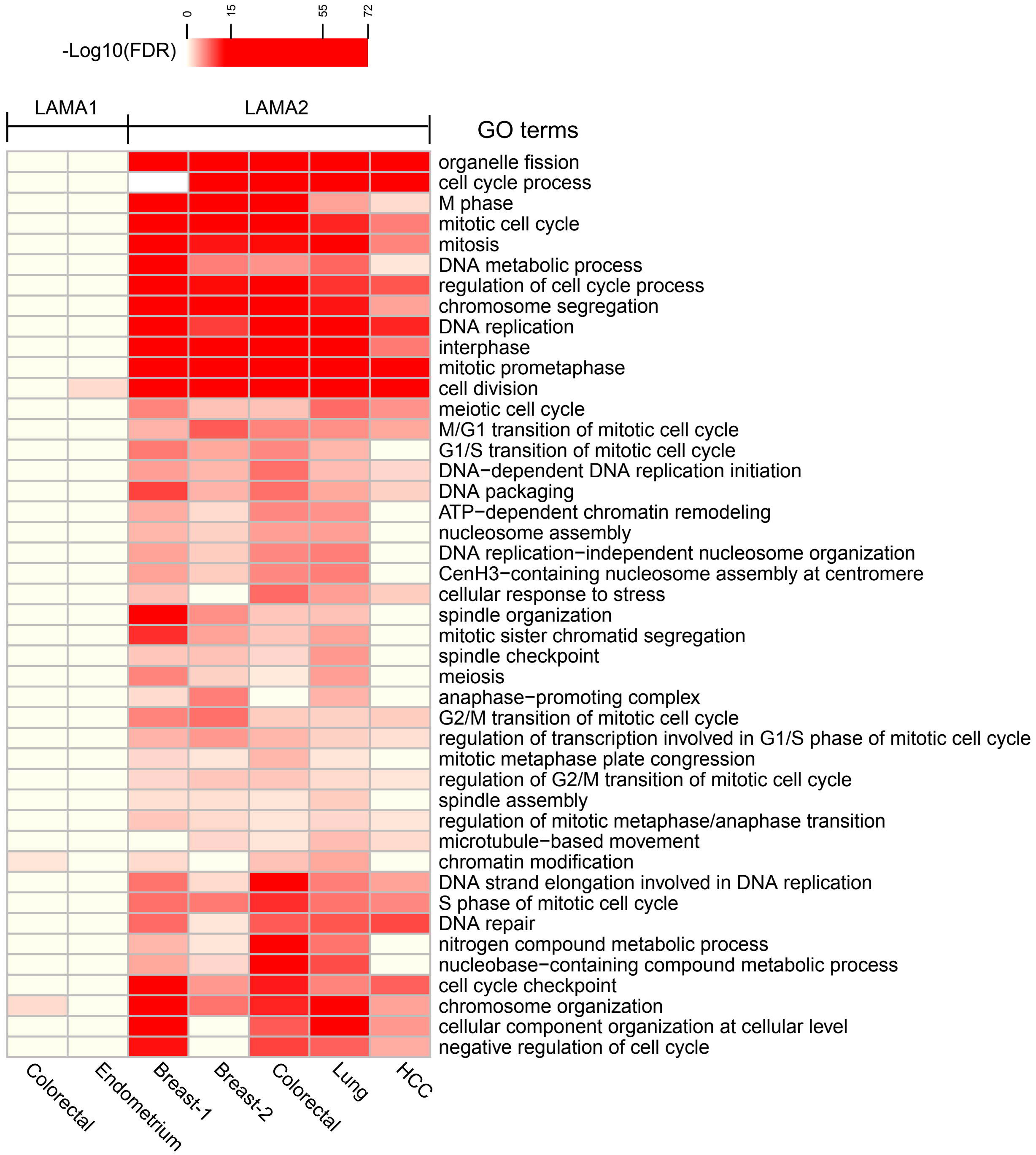

Figure S11. *LAMA2* down-regulation is associated with cell cycle regulation. We took the top (n = 500) up-regulated genes from *LAMA2*-low tumor groups from expression data across multiple cancer types and performed a GOstats analysis. We found a significant overlap of these genes with GO terms that are related to cell cycle control. A similar analysis performed on a different LAMA family member, *LAMA1*, did not show an enrichment of these GO terms. Colorectal, endometrium, breast-2 and lung cancer data are from the Genelogic microarray expression database. Breast-1 data is from The Cancer Genome Atlas (TCGA). HCC data is from GEO series GSE25097. FDR: false discovery rate (Benjamini-Hochberg adjusted p-values).
